# Ectopic breast cancer in vulva and other non-axillary sites: a systematic review

**DOI:** 10.1186/s12957-026-04358-8

**Published:** 2026-04-21

**Authors:** Kai Hui Loo, Mei-ju Hwang

**Affiliations:** 1 University of Edinburgh, Edinburgh, UK; 2 Department of General Surgery, Ysbyty Gwynedd, Betsi Cadwaladr University Health Board, Bangor, UK

**Keywords:** Ectopic breast cancer, Vulvar ectopic breast cancer, Non-axillary ectopic breast cancer, Extra-mammary breast cancer, Systematic review, Embryological remnants

## Abstract

**Background:**

Ectopic breast cancer (EBC) is a rare malignancy (0.3–0.6% of all breast cancers) that arises along the embryological milk line. While axillary EBC is better characterised and typically managed similarly to orthotopic breast cancer, the clinical features and management of non-axillary EBC remain poorly defined.

**Aim:**

To synthesise available data on the clinical presentation, histopathology, management, and outcomes of non-axillary EBC to refine treatment strategies.

**Methods:**

A systematic review of case reports and case series was conducted following PRISMA 2020 guidelines and registered with PROSPERO (CRD420251035771). PubMed, Ovid MEDLINE, Embase, Scopus, and Cochrane Library were searched up to April 2025. Study quality was assessed using the JBI checklist. Data were analysed descriptively using Microsoft Excel, focusing on clinical presentation, diagnosis, histopathology, treatment, and outcomes.

**Results:**

Sixty-one studies (63 patients) were included: 37 vulvar and 26 non-vulvar cases. Median age at diagnosis was 62. Lesions typically presented as painless nodules, with ulceration prompting attention. Invasive ductal adenocarcinoma was the most common histological subtype (39.7%), and oestrogen receptor positivity (82.5%) was frequent. Lymph node involvement was reported in 50.8% of cases. Organ metastases commonly involved bone, lung, and liver. Surgery was the main treatment, with adjuvant systemic therapy. Among cases with available follow-up (*n =* 50), recurrence and mortality rates were 18.0% (*n =* 9) and 16.0% (*n =* 8), respectively. Follow-up methods were poorly documented.

**Conclusion:**

Non-axillary EBC shares clinical and pathological characteristics with orthotopic breast cancer. Multidisciplinary, site-adapted approach is recommended with applying breast cancer principles to systemic management. Increased clinical awareness and more consistent reporting are crucial to optimise future care and outcomes.

**Supplementary Information:**

The online version contains supplementary material available at 10.1186/s12957-026-04358-8.

## Background

Ectopic breast tissue (EBT), first described by Hartung in 1872, is also known as supernumerary or aberrant breast tissue. It refers to breast tissue found outside its normal anatomical location on the anterior chest wall between the second and sixth ribs. Typically developing along the embryonic milk line from the axilla to the groin, EBT occurs in up to 6% of women [1, 2]. Fama et al. noted that ectopic tissue is capable of responding to hormonal influences similarly to orthotopic breast tissue, and may give rise to pathologies such as fibroadenomas and phyllodes tumours [3]. Ectopic breast cancer (EBC) is a rare malignancy that arises from EBT, accounting for approximately 0.3–0.6% of all breast cancer cases. The axilla is the most common site of involvement, representing nearly two-thirds of all EBC cases [4].

Axillary breast cancers are well described in the literature and are generally managed according to standard breast cancer guidelines. In contrast non-axillary EBC (e.g. vulvar, chest wall, groin, and perineum) remains poorly understood. These atypical presentations often mimic benign conditions including lipomas, cysts, or lymphadenopathy, leading to diagnostic delays and poor management outcomes. Furthermore, the absence of standardised diagnostic and therapeutic pathways contributes to significant variation in clinical practice.

There is no clear consensus on how to treat non-axillary EBC. Existing knowledge is limited primarily to isolated case reports and small case series, resulting in fragmented and inconsistent evidence. Additionally, outcome data are frequently underreported, limiting the ability to draw significant conclusions about optimum treatment and prognosis.

In this context, improved understanding of non-axillary EBC is essential. This systematic review aims to synthesis the current evidence of clinical presentation, diagnostic evaluation, and management strategies of non-axillary EBC. By addressing this gap in the literature, the review aims to contribute to consistent evidence-based clinical decision-making.

## Methods

This systematic review was conducted in accordance with PRISMA guidelines and registered with PROSPERO (CRD420251035771), available at: https://www.crd.york.ac.uk/PROSPERO/view/CRD420251035771

### Search strategy

A systematic literature search was conducted to identify studies reporting on non-axillary ectopic breast cancer (EBC). The search was conducted in accordance with the Preferred Reporting Items for Systematic Reviews and Meta-Analyses (PRISMA 2020) guidelines [5].

The following databases were searched from inception to April 2025: PubMed, Ovid (MEDLINE, Embase, AMED), Scopus, and the Cochrane Library. Search strategies combined controlled vocabulary (MeSH), Boolean operators (AND/OR), and free-text keywords: "breast cancer", "breast carcinoma", "ectopic breast", "supernumerary breast", and "polymastia". Terms related to axillary and accessory breast tissue were excluded. Complete search strategies are detailed in Supplementary Appendix 1.

All citations were exported into Rayyan (rayyan.ai), an online systematic review screening tool, where filters were applied to include English-language, human studies, and full-text availability. In total, 2,484 records were identified through database searches, and an additional 27 studies were identified via manual citation screening of included articles. Duplicate records were removed using Rayyan’s automated detection tool, with manual confirmation.

### Eligibility criteria

#### Inclusion criteria

Studies were included if they:

- Reported primary ectopic breast cancer located in non-axillary sites (vulva, chest wall, abdominal wall, groin).
- Provided details on clinical presentation, diagnostic methods, treatment, or follow-up.
- Were available in full text, in English, and involved human subjects.
- Classified as case reports and case series.

### Exclusion criteria

Studies were excluded if they involved:

- Axillary accessory breast tissue involvement.
- Synchronous orthotopic breast cancer.
- Metastatic breast cancer to ectopic sites (eg. secondary spread to the vulva or chest wall).
- Benign ectopic breast lesions, such as fibroadenoma, cysts, or hyperplasia.
- Non-human or non-English studies.
- Abstract-only publications or studies for which full text was not accessible.

Narrative reviews were screened for reference mining only, but no data were extracted from them to avoid duplication of reported cases.

#### Rationale for exclusion

Axillary EBC were excluded as these cases follow standard breast cancer treatment protocols due to their anatomical proximity to normal breast tissue. The review focused on the rarer locations of EBC due to their diagnostic and management complexity. Exclusion of synchronous or metastatic cases ensured analysis focused on primary malignancies arising from ectopic mammary tissue. Benign lesions were also excluded to limit the scope to malignancies.

### Study selection

Study selection was initially conducted by a single reviewer (KL), who performed title and abstract screening followed by full-text eligibility assessment according to predefined inclusion and exclusion criteria using Rayyan. To enhance methodological rigour, decisions regarding study inclusion and exclusion were reviewed and discussed with a second reviewer (MH), with consensus reached in cases of uncertainty. While formal dual independent screening was not performed, this approach provided an additional level of verification to minimise selection bias. In cases of ambiguity, decisions were made conservatively in favour of inclusion at the full-text stage.

Where full-text articles were not readily accessible through institutional subscriptions, assistance was sought from the medical librarian to obtain copies via inter-library loan services. If the full text remained unavailable by 16 April 2025, the study was excluded in accordance with the eligibility criteria.

### Data extraction

Data were manually extracted using Microsoft Excel and a standardised extraction form was developed to ensure consistency.

The following variables were collected:

- Author and year of publication
- Patient demographics
- Anatomical site of ectopic breast cancer
- Imaging modalities used
- Histopathological characteristics
- Hormone receptor status (ER, PR, HER2)
- Management strategies (surgery, radiotherapy, systemic therapy)
- Lymph node involvement
- Presence of distant metastases
- Follow-up duration and clinical outcomes

Where information was missing, unclear, or not reported in the original articles, a dash (–) was used in the data extraction table to denote the absence of available data.

### Data synthesis and analysis

Given the rarity of non-axillary EBC and the predominance of case reports, a narrative synthesis was conducted. Data were summarised qualitatively to identify patterns in clinical presentation, diagnostic methods, treatment strategies, and outcomes. Descriptive statistics were used: categorical variables were presented using frequencies and percentages, and age (the only continuous variable) was reported using mean, standard deviation (SD), median, and range. Due to the heterogeneity and limited number of cases, statistical comparisons and effect measures were not applied. All analyses were performed using Microsoft Excel. Formal tools for assessing certainty of evidence and risk of bias due to missing results were not used, as these are not applicable for narrative data. Follow-up duration was only recorded when explicitly reported. Cases without a specified follow-up duration were excluded from analysis of median follow-up. Recurrence and mortality rates are calculated based on cases with available follow-up data. When data permitted, descriptive subgroup analyses were conducted to compare outcomes by anatomical site, lymph node status and presence of distant metastasis. Results were presented in tabular format, with subgroup analyses summarised in the main text and detailed case-level data provided in supplementary tables.

### Quality assessment of included studies

The quality of the included studies was assessed using the Joanna Briggs Institute (JBI) Critical Appraisal Checklist for Case Reports [6]. Each study was reviewed using the eight-item tool, with ratings based on the number of criteria met. Items marked as “Not Applicable” were excluded from the total score. Studies were categorised as having low, moderate, or high risk of bias.

## Results

### Study selection

**Fig. 1:**
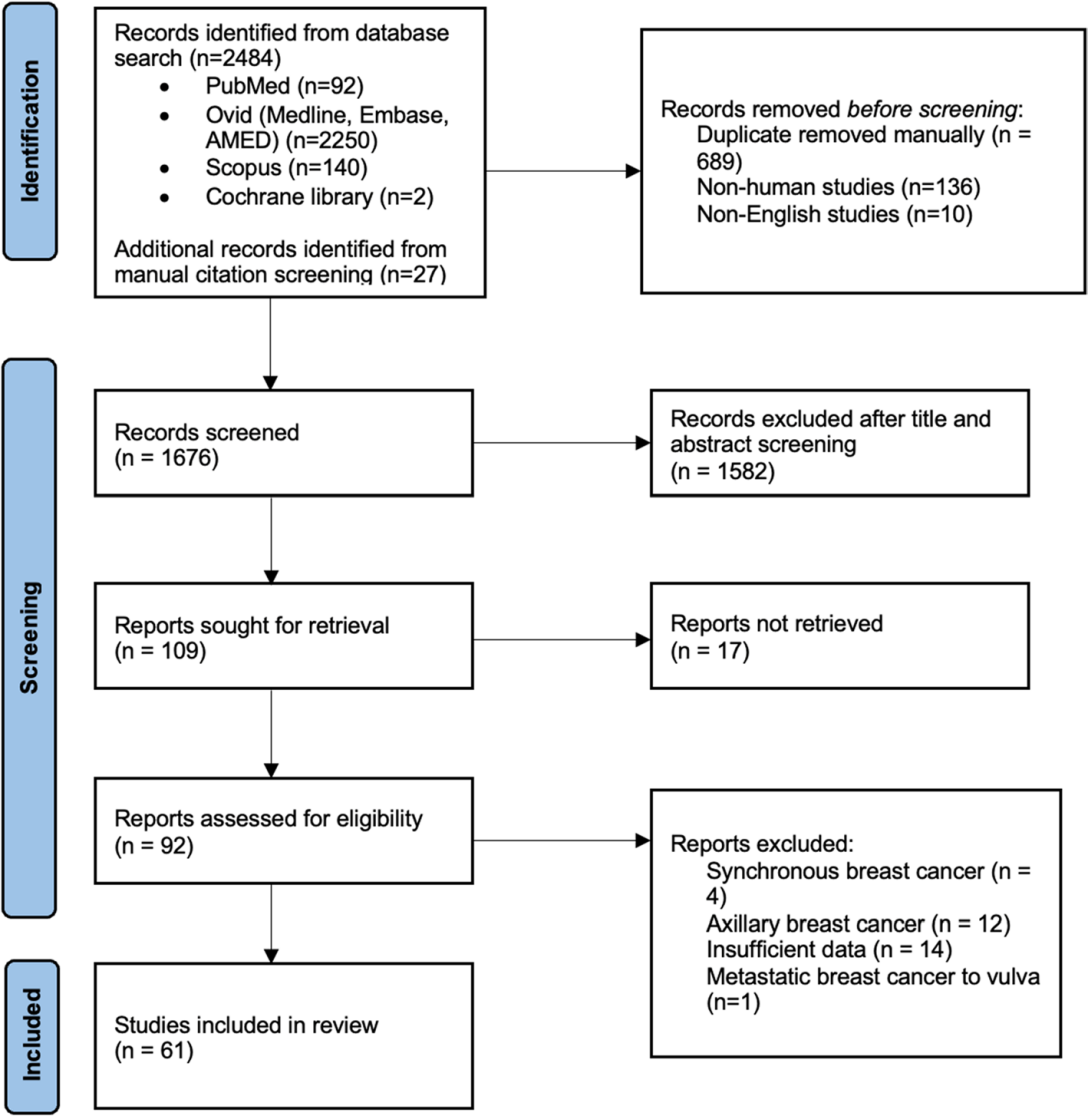
PRISMA flow diagram of the study selection process [5]. Source: Page MJ. BMJ 2021;372:n71. 10.1136/bmj.n71

A PRISMA flow diagram illustrating the study selection process is presented in Fig. 1. Initial database searches yielded 2,484 records, broken down as follows:

- PubMed: 92
- Ovid (MEDLINE, Embase, AMED): 2,250
- Scopus: 140
- Cochrane Library: 2

After removing 689 duplicates, 1,795 unique records remained. A further 146 records were excluded (136 non-human and 10 non-English studies), leaving 1,649 for title and abstract screening. An additional 27 records were identified through manual citation screening, resulting in a total of 1,676 records screened at this stage. Screening was conducted by a single reviewer using the Rayyan platform.

Following title and abstract screening, 109 full-text articles were retrieved for eligibility assessment. Of these, 61 studies met the inclusion criteria and were included in the qualitative synthesis.

Thirty-one studies were excluded at the full-text stage for the following reasons:

- Axillary accessory breast involvement (*n =* 12)
- Synchronous breast cancer (*n =* 4)
- Metastatic breast cancer (*n =* 1)
- Incomplete data (*n =* 14)

An additional 17 studies remained potentially eligible, pending full-text retrieval. Despite efforts to obtain these through inter-library requests, the literature search was concluded on 16 April 2025, and these studies were excluded due to unavailability. The excluded studies spanned publication years from 1947 to 2019, with a proportion originating from earlier decades where archival access was limited.

A total of 63 patients from 61 publications (60 single case reports and 1 three-patient case series) were included. The majority of cases were vulvar in origin (*n =* 37), with the remainder arising from other non-axillary anatomical sites (*n =* 26). Detailed clinicopathological characteristics, management and outcomes are provided in Supplementary Table S2 (vulvar cases) and Supplementary Table S3 (non-vulvar cases).

### Patient demographics

The mean age across all cases was 63 years (SD: 11.4), with a median of 62 years and an age range of 43 to 90 years. No significant difference in age distribution was observed between vulvar and non-vulvar cases.

### Clinical presentation

#### Vulvar ectopic breast cancer

A total of 37 vulvar ectopic breast cancer (EBC) cases were identified. Most lesions presented as nodules or masses, often asymptomatic initially, later becoming symptomatic due to growth, ulceration, bleeding, or pain. Summary statistics for lesion size and asymptomatic duration could not be calculated, as these variables were inconsistently reported. Palpable lymphadenopathy at presentation was noted in 5 cases (13.5%).

#### Non-vulvar ectopic breast cancer

A total of 26 non-vulvar EBC cases were identified. The most reported site was chest wall, in which lesions were frequently located at the inframammary fold, reported in 8 of 15 cases (53.3%), with accessory nipples observed in 6 cases (40.0%). Palpable lymphadenopathy was noted in 3 non-vulvar cases (11.5%), involving axillary, supraclavicular, inguinal, and pelvic nodes.

### Diagnostic investigation

Most cases underwent tissue diagnosis via excisional or incisional biopsy, or fine needle aspiration cytology (FNAC).

Breast imaging to exclude a primary breast lesion was performed in 47 out of 63 cases (74.6%), including mammography (*n =* 43) and breast ultrasonography and/or MRI (*n =* 4) where mammography was not conducted.

Following tissue diagnosis, additional imaging was employed for staging or to confirm the primary tumour site. These included:

- Computed tomography (CT): 38 cases
- Ultrasound (US): 32 cases
- Magnetic resonance imaging (MRI): 20 cases
- Positron emission tomography (PET-CT): 19 cases
- Chest X-ray (CXR): 14 cases
- Endoscopy/colonoscopy: 9 cases

Rare imaging methods included barium enema, thyroid scan, and diagnostic laparoscopy.

### Histopathology

The predominant histological subtype across all non-axillary EBC cases was invasive ductal adenocarcinoma (*n =* 25, 39.7%), followed by mammary-like invasive adenocarcinoma (*n =* 23, 36.5%), mucinous adenocarcinoma (*n =* 6, 9.5%), invasive lobular adenocarcinoma (*n =* 4, 6.4%), ductal carcinoma in situ (DCIS) (*n =* 4, 6.4%), and mammary-type tubulolobular carcinoma (*n =* 1, 1.6%).

Regarding hormone receptor status, most cases were oestrogen receptor-positive (ER +) (*n =* 52, 82.5%) and progesterone receptor-positive (PR +) (*n =* 43, 68.3%). HER2 overexpression was reported in 6 cases (9.5%). Two triple-negative EBC cases were identified, located in the vulva and scrotum, respectively.

### Lymph node involvement and organ metastasis

#### Vulvar ectopic breast cancer

Lymph node (LN) involvement was documented in 21 out of 37 vulvar EBCs (56.8%), most frequently affecting the superficial and deep inguinal LN. Organ metastases were reported in 6 cases (16.2%), most frequently involving the bone (*n =* 5), followed by the lung (*n =* 2), liver (*n =* 1), and skin (*n =* 1).

#### Non-vulvar ectopic breast cancer

LN involvement was identified in 11 of 26 non-vulvar EBCs (42.3%). Among chest wall cases with nodal metastases, the axillary nodes were most frequently affected (5 of 6), with additional involvement of subpectoral, intercostal, and supraclavicular nodes. Abdominal wall lesions demonstrated varied lymphatic spread, including to axillary, retroperitoneal, pelvic, bilateral inguinal, and peritoneal nodes. Inguinal LN involvement was also observed in perineal and scrotal EBCs. One suprapubic EBC reported pulmonary LN involvement at recurrence (likely representing intrathoracic nodal metastasis). Distant organ metastases were reported in 4 non-vulvar cases (15.4%), affecting the liver (*n =* 3), bone (*n =* 2), and lung (*n =* 1).

### Management and outcome

#### Surgical management

Primary tumour surgery was performed in 32 out of 37 vulvar cases (86.5%), comprising wide local excision (*n =* 12), partial vulvectomy (*n =* 7), radical vulvectomy (*n =* 12), and Mohs micrographic surgery (*n =* 1).

Lymph node (LN) surgery was performed in 25 vulvar cases (67.6%). Sentinel lymph node biopsy (SLNB) was attempted in 10 cases, with 2 requiring conversion to full dissection due to procedural failure and nodal involvement. Other LN procedures included unilateral (*n =* 6) and bilateral (*n =* 7) inguino-femoral dissection, and pelvic node dissection (*n =* 2).

Among non-vulvar cases, surgery was performed in 23 of 26 cases (88.5%). This included local excision (*n =* 8), wide local excision (*n =* 13), and ectopic breast resection (*n =* 2).

LN surgery was performed in 13 non-vulvar cases (50.0%). SLNB was performed in 8 cases, with 3 proceeding to axillary dissection due to nodal involvement (all chest wall). Five underwent direct LN dissection—targeting axillary (chest wall), or inguinal regions (perineal, inguinal, or scrotal sites).

#### Adjuvant and systemic therapy

Radiotherapy was administered in 25 out of 62 cases (40.3%), with a nearly equal distribution between vulvar (*n =* 13) and non-vulvar (*n =* 12) EBC. In vulvar cases, it typically targeted the primary site, inguinal, and pelvic nodes.

Chemotherapy was administered in 32 out of 63 patients (50.8%), comprising 18 vulvar and 14 non-vulvar cases, and was used across adjuvant, neoadjuvant, and palliative settings. Chemotherapy regimens appeared to be based on standard breast cancer protocols.

Endocrine or targeted therapy was administered in 41 of 63 patients (65.1%), including 25 vulvar and 16 non-vulvar cases. In the vulvar group, tamoxifen (*n =* 12) and aromatase inhibitors (AIs; *n =* 11) were commonly used. Two patients with ER +/HER2 + tumours received AIs with trastuzumab. In the non-vulvar group, treatments included tamoxifen (*n =* 6), AIs (*n =* 8), and unspecified agents (*n =* 2).

### Follow-up and outcomes

Follow-up protocols were inconsistently reported across the included studies, with 13 cases lacking any documented follow-up. Among the remaining 50 cases, reported methods included clinical examination, mammography, CT, bone scan and PET-CT.

Several studies indicated that follow-up was ongoing at the time of publication, and no cases reported formal discharge from surveillance. Follow-up duration was available in 42 of 63 cases (25/37 vulvar cases and 17/26 non-vulvar cases) and median values were calculated only from cases with explicitly stated follow-up periods. The overall median follow-up was 22 months. When analysed by subgroup, median follow-up was 24 months in vulvar cases (*n =* 25) and 17 months in non-vulvar cases (*n =* 17).

Outcome data were available in 28 of 37 vulvar cases and 22 of 26 non-vulvar cases. Overall, among cases with available follow-up (*n =* 50), recurrence occurred in 9 patients (18.0%), and 8 deaths (16.0%) were reported. Among vulvar cases, recurrence occurred in 5 patients (17.9%), and 6 deaths (21.4%) were reported. In non-vulvar cases, recurrence occurred in 4 patients (18.2%), and 2 deaths (9.1%) were reported. Recurrence and mortality rates were calculated only from cases with reported follow-up outcomes.

A subgroup comparison of outcomes between vulvar and non-vulvar cases is summarised in Table 1.

**Table 1:** Subgroup comparison of outcomes between vulvar EBC and non-vulvar EBC

Outcomes	Vulvar EBC (*n =* 37)	Non-Vulvar EBC (*n =* 26)
Lymph node involvement (%)	56.8% (21/37)	42.3% (11/26)
Organ metastasis (%)	16.2% (6/37)	15.4% (4/26)
Surgery performed (%)	86.5% (32/37)	88.5% (23/26)
Recurrence (%)	17.9% (5/28)	18.2% (4/22)
Mortality (%)	21.4% (6/28)	9.1% (2/22)

Recurrence and mortality rates are calculated based on cases with available outcome data

To further explore prognostic factors, outcomes were analysed according to lymph node status and summarised in Table 2. Recurrence occurred in 9 of 32 node-positive cases (28.1%), with no recurrences reported in node-negative cases. Mortality was 18.8% in node-positive and 4.3% in node-negative cases.

**Table 2:** Outcomes according to lymph node status

Outcomes	Node-positive (*n =* 32)	Node negative (*n =* 23)
Recurrence	28.1% (9/32)	0.0% (0/23)
Mortality	18.8% (6/32)	4.3% (1/23)

Recurrence and mortality rates are calculated based on cases with available outcome data

Outcomes by metastatic status are summarised in Table 3. Recurrence and mortality were higher in metastatic cases (55.6% and 44.4%, respectively) compared to non-metastatic cases (both 8.1%).

**Table 3:** Outcomes according to metastatic status

Outcomes	Metastasis (*n =* 9)	No metastasis (*n =* 37)
Recurrence	55.6% (5/9)	8.1% (3/37)
Mortality	44.4% (4/9)	8.1% (3/37)

Recurrence and mortality rates are calculated based on cases with available outcome data

### Quality assessment

The risk of bias in all included studies was assessed using the JBI Checklists for Case Reports [6]. Detailed JBI critical appraisal scores for each included study are presented in Supplementary Table S4.

A total of 63 cases were assessed. Based on adjusted scoring, 35 (55.5%) were classified as low risk of bias, 20 (31.7%) as moderate risk, and 8 (12.7%) as high risk.

Most studies clearly reported patient demographics and clinical presentation. However, a few focused narrowly on sentinel node techniques or histopathology, limiting information on interventions and outcomes. Ethnicity was underreported in 38 cases (60.3%), precluding any ethnicity-related analysis. Imaging, biopsy methods, and histopathology were consistently described. Hormonal receptor status (ER, PR, HER2) was documented in 60 cases (95.2%), with missing data mostly from older studies predating routine profiling.

Several included studies had incomplete clinical or outcome data, leading to a higher risk of bias, limiting their contribution to outcome analysis. Among the 13 cases reporting recurrence and/or mortality, the majority were derived from studies assessed as low risk of bias (*n =* 10), with only a minority from moderate-risk studies (*n =* 3).

## Discussion

Axillary ectopic breast cancer (EBC) is the most frequently reported subtype. Several reviews have proposed that axillary EBC be treated using standard breast cancer protocols due to its anatomical proximity and shared lymphatic drainage [3, 7]. However, the management of non-axillary EBC—particularly in sites such as the vulva, perineum, and chest wall—remains less well defined. This systematic review aimed to address this gap by evaluating the clinical characteristics and treatment approaches of vulvar and non-vulvar non-axillary EBC.

The median age at presentation was 62 years, aligning with the typical age range for breast cancer. The gender distribution was notably skewed (56 females vs. 7 males), primarily due to the high proportion of vulvar cases and the exclusion of axillary EBC. Interestingly, Marshall et al. (reviewing all EBC cases) and Visconti et al. (focusing on axillary EBC) reported a comparatively higher male representation than is typically observed in orthotopic breast cancer [7, 8].

Most lesions were slow-growing, painless nodules that remained asymptomatic for years, with some unrecognised accessory breast present since childhood. Clinical attention was often prompted by ulceration or pain, frequently following initial misdiagnosis as benign conditions. This diagnostic delay occasionally led to inoperable disease.

Lymph node involvement was reported in 50.8% of non-axillary EBC cases, similar to the 60% by Abbott et al., and higher than the 29% seen in orthotopic breast cancer and approximate 30% in operable vulvar cancer [9–11]. Similar high rates were also reported for axillary EBC, with 51.8% by Nihon-Yanagi et al. and 46% by Marshall et al. Although some attribute this to proximity to axillary nodes, Nihon-Yanagi et al. found no correlation between tumour size and nodal metastasis, leaving the cause unclear [8, 12]. Despite anatomical variation, EBC showed similar metastatic patterns to orthotopic breast cancer, most commonly involving bone, lung, and liver [13].

The relative high rate of nodal involvement observed in this review should be interpreted in the context of potential reporting bias, as case reports are more likely to describe advanced or clinically significant presentations. While delayed diagnosis may contribute, this cannot be confirmed from the available data.

Histologically, invasive ductal adenocarcinoma was the most reported subtype (39.7%), followed closely by mammary-like invasive adenocarcinoma (37.1%). While these were recorded separately due to distinct terminology used in the original case reports, mammary-like adenocarcinomas share key morphological and immunohistochemical features with invasive ductal carcinoma [14]. Mucinous carcinoma was exclusively identified in vulvar lesions. Most tumours were ER-positive and PR-positive, whereas HER2 positivity was less common. Overall, these histological and receptor profiles align with patterns seen in orthotopic breast cancer [15]. However, this similarity should not be interpreted as definitive biological equivalence. Evidence remains largely descriptive with limited molecular data and unclear role of the local tissue microenvironment in influencing tumour behavior. Therefore, while parallels can be drawn, the extent to which EBC truly mirror conventional breast carcinoma remains uncertain.

Surgical resection of the primary lesion remained the mainstay treatment. In this review, nearly one third of the vulvar EBC cases underwent bilateral lymph node dissections, in contrast with current vulvar cancer guidelines recommending less invasive approaches—reserving bilateral dissection for midline tumours and SLNB for lateralised lesions < 4 cm [16]. As no specific guidelines exist for vulvar EBC, vulvar cancer guidelines may serve as a reference for surgical planning. This approach may reduce morbidity associated with inguinal node dissection [17]. Notably, several patients underwent bilateral dissections without meeting criteria but were later found to have nodal involvement, raising the possibility that vulvar EBC may follow different lymphatic risk patterns [2, 18–28].

For chest wall lesions, lymphatic spread mirrored that of orthotopic breast cancer, supporting similar management strategies. Management of ectopic breast tissue, especially in the axilla and chest wall, remains debated. Roorda et al. and Bi et al. advocate prophylactic removal due to its non-functional nature and malignant potential [29, 30]. Other authors suggest that the orthotopic breast should be preserved in the absence of malignancy [7, 31, 32]. Based on this rationale, we hypothesise that removing ectopic breast tissue may be appropriate, whereas prophylactic mastectomy of the orthotopic breast does not appear to be justified based on current limited evidence. In our review, two chest wall EBC cases involved ectopic tissue resection, based on case-specific decisions.

Radiotherapy was administered in 41.3% of non-axillary EBC cases, reflecting variable practice patterns likely influenced by tumour location and nodal involvement. Given the lack of specific guidance for vulvar EBC, management approaches extrapolated from vulvar cancer protocols may be considered, although this should be interpreted cautiously as a provisional strategy given biological differences and the absence of comparative outcome data. A randomised controlled trial by Kunos et al. recommended postoperative pelvic and inguinal radiotherapy for vulvar cancer patients with inguinal node metastases, which may be similarly applicable in cases of nodal involvement in vulvar EBC [33].

Chemotherapy was used in 50.8% of non-axillary EBC cases, with regimens commonly mirroring those used in breast cancer [34]. Endocrine therapy was administered in 65.1% of patients, primarily using tamoxifen or aromatase inhibitors, consistent with standard breast cancer protocols.

Follow-up reporting was inconsistent, with 13 studies lacking post-treatment outcome data. Given the heterogeneity of treatment among recurrence cases, no clear treatment pattern could be associated with recurrence. Due to the small sample size, inconsistent reporting, and limited long-term follow-up data, reliable estimation of 5-year survival or mortality was not feasible in this review. Similar to other EBC reviews, long-term prognosis remains difficult to establish. Several studies have suggested that poorer outcomes in EBC may reflect delays in diagnosis and management rather than the inherent aggressiveness of the disease [7, 8, 12].

The potential impact of excluding studies due to full-text unavailability should be considered. Although a proportion of these reports originated from earlier decades, their exclusion may still influence the findings given the small overall sample size and rarity of the condition. Therefore, the results should be interpreted with caution.

This review emphasises the clinical and pathological similarities between non-axillary EBC and orthotopic breast cancer, suggesting the appropriateness of standard breast cancer protocols for systemic management. For vulvar EBC, in the absence of site-specific guidelines, the adoption of existing vulvar cancer protocols for surgical and radiotherapeutic management is recommended, pending future evidence to support alternative approaches. For non-vulvar, non-axillary EBC treatment may be individualised, guided by anatomical considerations and extrapolated from breast cancer principles. Follow-up may involve site-relevant specialties, such as gynaecologic oncology for vulvar cases. As no current evidence links EBC to a higher risk of subsequent primary breast cancer, decisions regarding mammographic surveillance should be guided by local multidisciplinary team discussion and existing protocols.

### Limitations

This systematic review has several limitations. Firstly, the included studies are predominantly case reports, which are inherently prone to recall and reporting bias. The study selection process was conducted by a single reviewer, which may introduce selection bias and reduce reproducibility compared to dual independent screening. Although study eligibility decisions were reviewed with a supervision author, the absence of formal independent duplicate screening may have increased the risk of missed studies and subjective inclusion decisions.

Despite a comprehensive search and manual screening, some studies were excluded due to unavailable full texts, potentially introducing publication and retrieval bias. The included studies may not fully capture the complete spectrum of reported cases. Limiting the review to English-language articles may have excluded relevant data, especially from countries like Japan and China where ectopic breast cancer has also been frequently reported.

Heterogeneity across studies was substantial, including differences in staging, receptor profiling, particularly in earlier reports where testing was incomplete or unavailable. Evolving diagnostic and systemic treatment strategies over time may influence reported outcomes. These factors limit comparability across studies and should be considered when interpreting the findings.

The limited follow-up and incomplete outcome reporting observed in higher-risk studies may result in under-reporting of recurrence and mortality. Lastly, the overall sample size remains small, reflecting the rarity of ectopic breast cancer, which limits the ability to draw generalisable conclusions or identify strong patterns in clinical behaviour, management, or prognosis.

## Conclusions

This review highlights the diagnostic and management challenges associated with non-axillary ectopic breast cancer. Despite anatomical differences, the biological behaviour and treatment response of these tumours closely mirror those of conventional breast cancer. A multidisciplinary, site-specific approach, guided by existing breast and gynaecological oncology protocols, appears appropriate. Greater clinical awareness, the development of clearer management guidelines, and more consistent outcome reporting are essential to optimise care and guide future research efforts. Establishing a minimum data set and promoting global data collection are crucial steps to guide future evidence-based care.

## Supplementary Information

Supplementary Material 1: Supplementary Table S1. PRISMA 2020 Checklist. Supplementary Appendix 1. Search Strategy. Supplementary Table S2. Summary of clinicopathological characteristics, treatment, and outcomes of vulvar ectopic breast cancer cases [2, 9, 18–28, 35–56]. Supplementary Table S3. Summary of clinicopathological characteristics, treatment, and outcomes of non-vulvar ectopic breast cancer cases [29, 57–81]. Supplementary Table S4. JBI critical appraisal scores for each included study across all checklist items.

## Data Availability

All data generated or analysed during this study are included in this published article and its supplementary information files.
