# Supplementary material for: Ectopic breast cancer in vulva and other non-axillary sites: a systematic review
Source: World J Surg Oncol. 2026 Apr 21;24:240. doi: 10.1186/s12957-026-04358-8 (PMC13227853; doi:10.1186/s12957-026-04358-8)
Supplement: Supplementary file 1 — Supplementary Material 1: Supplementary Table S1. PRISMA 2020 Checklist. Supplementary Appendix 1. Search Strategy. Supplementary Table S2. Summary of clinicopathological characteristics, treatment, and outcomes of vulvar ectopic breast cancer cases [2, 9, 18–28, 35–56]. Supplementary Table S3. Summary of clinicopathological characteristics, treatment, and outcomes of non-vulvar ectopic breast cancer cases [29, 57–81]. Supplementary Table S4. JBI critical appraisal scores for each included study across all checklist items. [file 12957_2026_4358_MOESM1_ESM.docx]

Supplementary Table S1: PRISMA 2020 Checklist

| **Section and Topic** | **Item #** | **Checklist item** | **Location where item is reported** |
| --- | --- | --- | --- |
| **TITLE** | | |  |
| Title | 1 | Identify the report as a systematic review. | Page 1 |
| **ABSTRACT** | | |  |
| Abstract | 2 | See the PRISMA 2020 for Abstracts checklist. | Page 2 |
| **INTRODUCTION** | | |  |
| Rationale | 3 | Describe the rationale for the review in the context of existing knowledge. | Page 3-4 |
| Objectives | 4 | Provide an explicit statement of the objective(s) or question(s) the review addresses. | Page 3-4 |
| **METHODS** | | |  |
| Eligibility criteria | 5 | Specify the inclusion and exclusion criteria for the review and how studies were grouped for the syntheses. | Page 5-6 |
| Information sources | 6 | Specify all databases, registers, websites, organisations, reference lists and other sources searched or consulted to identify studies. Specify the date when each source was last searched or consulted. | Page 5, 26-34, |
| Search strategy | 7 | Present the full search strategies for all databases, registers and websites, including any filters and limits used. | Page 5, 36 |
| Selection process | 8 | Specify the methods used to decide whether a study met the inclusion criteria of the review, including how many reviewers screened each record and each report retrieved, whether they worked independently, and if applicable, details of automation tools used in the process. | Page 7 |
| Data collection process | 9 | Specify the methods used to collect data from reports, including how many reviewers collected data from each report, whether they worked independently, any processes for obtaining or confirming data from study investigators, and if applicable, details of automation tools used in the process. | Page 7 |
| Data items | 10a | List and define all outcomes for which data were sought. Specify whether all results that were compatible with each outcome domain in each study were sought (e.g. for all measures, time points, analyses), and if not, the methods used to decide which results to collect. | Page 8 |
|  | 10b | List and define all other variables for which data were sought (e.g. participant and intervention characteristics, funding sources). Describe any assumptions made about any missing or unclear information. | Page 8 |
| Study risk of bias assessment | 11 | Specify the methods used to assess risk of bias in the included studies, including details of the tool(s) used, how many reviewers assessed each study and whether they worked independently, and if applicable, details of automation tools used in the process. | Page 9, 41-43 |
| Effect measures | 12 | Specify for each outcome the effect measure(s) (e.g. risk ratio, mean difference) used in the synthesis or presentation of results. | N/A |
| Synthesis methods | 13a | Describe the processes used to decide which studies were eligible for each synthesis (e.g. tabulating the study intervention characteristics and comparing against the planned groups for each synthesis (item #5)). | Page 8 |
|  | 13b | Describe any methods required to prepare the data for presentation or synthesis, such as handling of missing summary statistics, or data conversions. | Page 8 |
|  | 13c | Describe any methods used to tabulate or visually display results of individual studies and syntheses. | Page 8-9 |
|  | 13d | Describe any methods used to synthesize results and provide a rationale for the choice(s). If meta-analysis was performed, describe the model(s), method(s) to identify the presence and extent of statistical heterogeneity, and software package(s) used. | Page 8 |
|  | 13e | Describe any methods used to explore possible causes of heterogeneity among study results (e.g. subgroup analysis, meta-regression). | Page 8 |
|  | 13f | Describe any sensitivity analyses conducted to assess robustness of the synthesized results. | N/A |
| Reporting bias assessment | 14 | Describe any methods used to assess risk of bias due to missing results in a synthesis (arising from reporting biases). | Page 9, 41-43 |
| Certainty assessment | 15 | Describe any methods used to assess certainty (or confidence) in the body of evidence for an outcome. | N/A |
| **RESULTS** | | |  |
| Study selection | 16a | Describe the results of the search and selection process, from the number of records identified in the search to the number of studies included in the review, ideally using a flow diagram. | Page 9-11 |
|  | 16b | Cite studies that might appear to meet the inclusion criteria, but which were excluded, and explain why they were excluded. | Page 10 |
| Study characteristics | 17 | Cite each included study and present its characteristics. | Page 11, 37-40 |
| Risk of bias in studies | 18 | Present assessments of risk of bias for each included study. | Page 17-18, 41-43 |
| Results of individual studies | 19 | For all outcomes, present, for each study: (a) summary statistics for each group (where appropriate) and (b) an effect estimate and its precision (e.g. confidence/credible interval), ideally using structured tables or plots. | N/A |
| Results of syntheses | 20a | For each synthesis, briefly summarise the characteristics and risk of bias among contributing studies. | Page 17-18 |
|  | 20b | Present results of all statistical syntheses conducted. If meta-analysis was done, present for each the summary estimate and its precision (e.g. confidence/credible interval) and measures of statistical heterogeneity. If comparing groups, describe the direction of the effect. | N/A (descriptive synthesis only) |
|  | 20c | Present results of all investigations of possible causes of heterogeneity among study results. | N/A |
|  | 20d | Present results of all sensitivity analyses conducted to assess the robustness of the synthesized results. | N/A |
| Reporting biases | 21 | Present assessments of risk of bias due to missing results (arising from reporting biases) for each synthesis assessed. | N/A, reporting bias discussed qualitatively in Discussion |
| Certainty of evidence | 22 | Present assessments of certainty (or confidence) in the body of evidence for each outcome assessed. | N/A |
| **DISCUSSION** | | |  |
| Discussion | 23a | Provide a general interpretation of the results in the context of other evidence. | Page 18-22 |
|  | 23b | Discuss any limitations of the evidence included in the review. | Page 22-23 |
|  | 23c | Discuss any limitations of the review processes used. | Page 22-23 |
|  | 23d | Discuss implications of the results for practice, policy, and future research. | Page 19-24 |
| **OTHER INFORMATION** | | |  |
| Registration and protocol | 24a | Provide registration information for the review, including register name and registration number, or state that the review was not registered. | Page 5 |
|  | 24b | Indicate where the review protocol can be accessed, or state that a protocol was not prepared. | Page 5 |
|  | 24c | Describe and explain any amendments to information provided at registration or in the protocol. | N/A |
| Support | 25 | Describe sources of financial or non-financial support for the review, and the role of the funders or sponsors in the review. | Page 24 |
| Competing interests | 26 | Declare any competing interests of review authors. | Page 24 |
| Availability of data, code and other materials | 27 | Report which of the following are publicly available and where they can be found: template data collection forms; data extracted from included studies; data used for all analyses; analytic code; any other materials used in the review. | Page 24, 36-43 |

Footnote: N/A, not applicable

*From:*  Page MJ, McKenzie JE, Bossuyt PM, Boutron I, Hoffmann TC, Mulrow CD, et al. The PRISMA 2020 statement: an updated guideline for reporting systematic reviews. BMJ 2021;372:n71. doi: 10.1136/bmj.n71. This work is licensed under CC BY 4.0. To view a copy of this license, visit <https://creativecommons.org/licenses/by/4.0/>

Supplementary Appendix 1: Search Strategy

PubMed

("Breast Neoplasms"[Mesh] OR "breast cancer"[tiab] OR "breast carcinoma"[tiab] OR "mammary cancer"[tiab]) AND ("Mammary Glands, Ectopic"[Mesh] OR "Ectopic Tissue"[Mesh] OR "ectopic breast"[tiab] OR "supernumerary breast"[tiab] OR "aberrant breast"[tiab] OR "polymastia"[tiab]) NOT ("Axilla"[Mesh] OR axilla[tiab] OR axillary[tiab] OR "accessory breast"[tiab])

Ovid MEDLINE

1. exp Breast Neoplasms/
2. breast cancer.tw. OR breast carcinoma.tw. OR mammary cancer.tw.
3. 1 OR 2
4. exp Mammary Glands, Ectopic/ OR exp Ectopic Tissue/
5. ectopic breast.tw. OR supernumerary breast.tw. OR aberrant breast.tw. OR polymastia.tw.
6. 4 OR 5
7. axilla.tw. OR axillary.tw. OR accessory breast.tw.
8. 3 AND 6
9. 8 NOT 7
10. limit 9 to (english language AND humans AND full text)

Cochrane Library

("breast cancer" OR "breast carcinoma" OR "mammary cancer") AND ("ectopic breast" OR "supernumerary breast" OR "aberrant breast" OR "ectopic tissue" OR "polymastia") NOT (axilla OR axillary OR "accessory breast")

Scopus

TITLE-ABS-KEY("breast cancer" OR "breast carcinoma" OR "mammary cancer") AND
TITLE-ABS-KEY("ectopic breast" OR "supernumerary breast" OR "aberrant breast" OR "ectopic tissue" OR "polymastia") AND

NOT TITLE-ABS-KEY(axilla OR axillary OR "accessory breast"

Supplementary Table S2: Summary of clinicopathological characteristics, treatment, and outcomes of vulvar ectopic breast cancer cases.

| **No** | **Author** | **Year** | **Age** | **Site** | **Histology** | **Hormonal status** | **Lymph node status** | **Organ metastasis** | **Surgery** | **Chemotherapy** | **Radiation therapy** | **Hormonal/targeted therapy** | **Complication/Follow-up/ Outcome** |
| --- | --- | --- | --- | --- | --- | --- | --- | --- | --- | --- | --- | --- | --- |
| 1 | Greene et al(7) | 1935 | 49 | Right labia | Breast adenocarcinoma | - | - | - | N | - | - | - | Died at 1 mo post-diagnosis. |
| 2 | Simon et al(8) | 1988 | 60 | Right labia majora | Breast adenocarcinoma | ER+, PR+ | Y | N | Y | Y | Y | Y | Recurrence at 6mo; died at 27mo. |
| 3 | Rose et al(9) | 1990 | 68 | Right vulvar | Invasive ductal adenocarcinoma | ER+ | Y | N | Y | - | Y | Y | Developed bilateral femoral deep vein thrombosis at 4weeks post-op. |
| 4 | Bailey et al(10) | 1993 | 65 | Right labia majora + minora | Invasive ductal adenocarcinoma | ER+, PR+ | Y | N | Y | - | - | Y | No recurrence at 1yr. |
| 5 | Levin et al(11) | 1995 | 62 | Left paraclitoral | Breast Adenocarcinoma | ER+ | Y | Y | Y | - | Y | Y | Recurrence at 2yr. No recurrence at 1yr post treatment. |
| 6 | Kennedy et al(12) | 1996 | 71 | Left labia majora | Invasive ductal adenocarcinoma | ER+, PR- | Y | N | Y | Y | Y | - | No recurrence at 1yr. |
| 7 | Irvin et al(2) | 1999 | 64 | Lateral anterior mon pubis | Mammary differentiation in poorly differentiated adenocarcinoma | ER+, PR+ | Y | N | Y | Y | Y | Y | - |
| 8 | Castro et al(13) | 2001 | 57 | Right labia majora | Ductal carcinoma in situ | ER+, PR+ | N | N | Y | - | - | - | No recurrence at 1yr. |
| 9 | Chung-Park et al(14) | 2002 | 47 | Right labia minor | Mucinous adenocarcinoma | ER+ | - | N | Y | - | - | - | F/up with annual mammogram. No recurrence up to 3yr. |
| 10 | Yin et al(15) | 2003 | 84 | Left vulva | Mucinous adenocarcinoma | ER+, PR+, HER2- | Y | N | Y | - | - | - | - |
| 11 | Tanaka et al(16) | 2005 | 87 | Left labia majora | Mammary-like adenocarcinoma | ER-, PR- | - | Y | N | Y | - | - | - |
| 12 | Lopes et al(17) | 2006 | 44 | Left vulvar | Mucinous carcinoma arising in ectopic mammary tissue | ER+, HER2- | Y | N | Y | Y | - | Y | New ectopic breast cancer at inguinal 19yrs later. |
| 13 | Fracchioli et al(18) | 2006 | 57 | Left vulvar | Invasive ductal adenocarcinoma | ER- | Y | Y | Y | Y | - | N | Developed left limb oedema. Recurrence and died at 3yr. |
| 14 | Abbott et al(19) | 2006 | 51 | Right labia majora | Adenocarcinoma arising in mammary-like gland | ER-, PR-, HER2-, (triple negative) | N | N | Y | - | - | N | No recurrence at 26mo. |
| 15 | North et al(20) | 2007 | 49 | Right anterior vulva | Invasive ductal adenocarcinoma | ER+, PR+, HER2- | Y | N | Y | Y | Y | Y | - |
| 16 | Martinez-palones et al(21) | 2007 | 49 | Right labia majora | Adenocarcinoma-like mammary gland | ER+, PR+ | N | N | Y | N | N | Y | - |
| 17 | Naseer et al(22) | 2011 | 57 | Right labia majora | Invasive ductal adenocarcinoma | ER+, PR+, HER2- | Y | N | Y | Y | Y | Y | F/up clinically. No recurrence. |
| 18 | da Costa et al(23) | 2012 | 82 | Left labia minor | Invasive adenocarcinoma of mammary origin | ER+, PR + | N | N | Y | N | Y | Y | No recurrence at 4yr. |
| 19 | Benito et al(24) | 2013 | 82 | Left labia | Invasive ductal adenocarcinoma | ER+, PR+ | Y | N | Y | N | N | Y | No recurrence at 2yr. |
| 20 | Bogani et al(25) | 2013 | 71 | Left labia majora | Breast carcinoma | ER+, PR+ | Y | N | Y | Y | N | Y | F/up clinically + mammogram. No recurrence at 2yr. |
| 21 | McMaster et al(26) | 2013 | 60 | Left vulva | Invasive adenocarcinoma | ER+ | N | N | Y | - | Y | - | - |
| 22 | Lamb et al(27) | 2013 | 59 | Left labia majora | Infiltrating mammary adenocarcinoma with mucinous differentiation | ER+, PR+, HER2- | N | N | Y | - | - | Y | - |
| 23 | Cripe et al(28) | 2015 | 62 | Left labial | Invasive ductal adenocarcinoma | ER+, PR+, HER2- | Y | N | Y | Y | Y | Y | Local recurrence at 3mo. No recurrence at 13mo post treatment. |
| 24 | Serre et al(29) | 2016 | 70 | Right labia majora | Invasive ductal adenocarcinoma | ER+, PR+ | Y | N | Y | Y | - | Y | No recurrence at 20mo. |
| 25 | Kredentser et al(30) | 2016 | 75 | Right labial | Adenocarcinoma from mammary tissue | ER+, PR-, HER2+ | Y | N | Y | Y | Y | Y | No recurrence at 5yr. |
| 26 | Kredentser et al(30) | 2016 | 59 | Right vulvar | Mucinous adenocarcinoma | ER-, PR+, HER2- | Y | N | Y | Y | Y | Y | No recurrence at 5yr. |
| 27 | Kredentser et al(30) | 2016 | 67 | Right labia majora | Mucinous adenocarcinoma | ER+, PR+ | N | N | Y | - | - | Y | No recurrence at 3yr. |
| 28 | Ishigaki et al(31) | 2017 | 72 | Right vulva | Invasive ductal adenocarcinoma | ER+, PR+, HER2- | N | N | Y | - | - | Y | No recurrence at 6mo. |
| 29 | Al-Mansouri et al(32) | 2018 | 76 | Pelvis | Poorly differentiated adenocarcinoma | ER+, PR+, HER2- | Y | N | N | Y | Y | Y | Recurrence and died at 2yr. |
| 30 | Lopes et al(33) | 2018 | 58 | Right vulva | Invasive carcinoma with mammary features | ER+, HER2+ | N | N | Y | Y | - | Y | - |
| 31 | Matak et al(34) | 2020 | 60 | Mons pubis | Invasive lobular adenocarcinoma | ER+, PR+ | N | N | Y | N | N | N | No recurrence at 10yr. |
| 32 | Ananthula et al(35) | 2020 | 47 | Right labia majora | Mammary-type adenocarcinoma | ER+, PR-, HER2- | Y | Y | Y | Y | N | Y | Died at 18mo. |
| 33 | Xie et al(36) | 2021 | 62 | Vulvar | Mammary-like carcinoma | ER+, PR+, HER2+ | Y | Y | N | Y | N | Y | F/up with CT. Stable disease at 18mo. |
| 34 | Kovacs et al(37) | 2022 | 68 | Vulvar | Ductal carcinoma in situ | ER+, PR+, HER2- | N | N | Y | - | Y | Y | No recurrence at 8yr. |
| 35 | Sandak et al(38) | 2023 | 51 | Left introitus | Ductal carcinoma in situ | ER+, PR+ | N | N | Y | - | - | Y | No recurrence at 4mo. |
| 36 | Aksu et al(39) | 2024 | 64 | Right labia majora | Breast Adenocarcinoma | ER+, PR+ | N | Y | N | Y | N | - | Died shortly after treatment commenced. |
| 37 | Tang et al(40) | 2024 | 69 | Vulvar | Invasive ductal adenocarcinoma | ER+, PR+, HER2+ | Y | N | Y | - | - | - | - |

**Footnote and abbreviations: ER**, oestrogen receptor; **PR**, progesterone receptor; **HER2**, human epidermal growth factor receptor 2; **+/−**, positive/negative receptor status; **Y**, yes; **N**, no; **−**, not mentioned in article; **N/A**, not applicable; **Mo**, month; **Yr**, year; **F/up**, follow-up; **Lymph node and** **Organ metastasis**, includes metastases present at diagnosis and at recurrence (recurrence time frame is specified when applicable)

Supplementary Table S3: Summary of clinicopathological characteristics, treatment, and outcomes of non-vulvar ectopic breast cancer cases.

| **No** | **Author** | **Year** | **Age** | **Sex** | **Site** | **Histology** | **Hormonal status** | **Lymph node status** | **Organ metastasis** | **Surgery** | **Chemotherapy** | **Radiation therapy** | **Hormonal/targeted therapy** | **Complication/Follow-up/**  **Outcome** |
| --- | --- | --- | --- | --- | --- | --- | --- | --- | --- | --- | --- | --- | --- | --- |
| 1 | Livesey et al(41) | 1990 | 43 | F | Left chest wall | Invasive ductal adenocarcinoma | - | Y | Y | Y | - | Y | Y | Recurrence at 6mo. |
| 2 | Dyess et al(42) | 1995 | 67 | F | Right chest wall | Invasive adenocarcinoma | ER+, PR- | N | - | Y | - | - | - | - |
| 3 | Rho et al(43) | 2001 | 61 | F | Right chest wall | Invasive poorly differentiated adenocarcinoma of mammary origin | - | - | - | Y | Y | Y | - | No recurrence at 15 mo. |
| 4 | Roorda et al(44) | 2002 | 70 | F | Left chest wall | Invasive ductal adenocarcinoma | ER+, PR+ | - | N | Y | - | Y | Y | No recurrence at 9yr. |
| 5 | Kazakov et al(45) | 2006 | 64 | F | perineal | Mammary-type tubulolobular carcinoma | ER+, PR+, HER2- | - | - | Y | - | - | - | No recurrence at 5.5yr |
| 6 | van Herwaarden-Lindeboom et al(46) | 2007 | 46 | F | Anterior chest wall | Invasive lobular adenocarcinoma | ER+, PR+, HER2- | Y | N | Y | Y | Y | Y | - |
| 7 | da Silva et al(47) | 2008 | 86 | F | Right chest wall | Invasive ductal adenocarcinoma | ER+, PR+ | Y | N | Y | - | N | N | Died at 3 mo. |
| 8 | Kahraman-cetintas et al(48) | 2008 | 49 | F | Right chest wall | Invasive lobular adenocarcinoma | ER-, PR+ | Y | N | Y | Y | Y | Y | No recurrence at 3.5yr. |
| 9 | Ogino et al(49) | 2010 | 70 | F | Left chest wall | Invasive ductal adenocarcinoma | ER+, PR+, HER2- | N | N | Y | N | N | Y | No recurrence at 1yr. |
| 10 | Rajan et al(50) | 2011 | 90 | F | Left chest wall | Ductal carcinoma in situ | ER+ | N | - | N | N | N | Y | F/up at 2mo, good clinical response with lesion halved in size |
| 11 | Francone et al(51) | 2013 | 43 | F | Right chest wall | Invasive ductal adenocarcinoma | ER+, PR+ | Y | N | Y | Y | - | Y | No recurrence at 8yr. |
| 12 | Xu et al(52) | 2015 | 59 | F | Anterior chest wall | Moderately differentiated adenocarcinoma | ER+, PR-, HER2- | N | N | Y | Y | Y | Y | No recurrence at 3.5yr. |
| 13 | Samanta et al(53) | 2015 | 60 | M | Right chest wall | Invasive ductal adenocarcinoma | ER+, PR+ | - | - | N | Y | - | - | Lost for f/up |
| 14 | Kopanakis et al(54) | 2016 | 58 | M | Umbilicus | Poorly differentiated carcinoma of mammary origin | ER+, PR+ | N | N | Y | Y | - | Y | No recurrence at 10mo. |
| 15 | Eom et al(55) | 2017 | 70 | M | Perineal | Invasive breast carcinoma | ER+, PR+, HER2- | Y | N | Y | Y | - | Y | No recurrence at 8mo. |
| 16 | Zhong et al(56) | 2018 | 62 | M | Right abdominal wall | Invasive ductal adenocarcinoma | ER+, PR+, HER2- | Y | N | Y | - | Y | Y | Stable disease at 6mo. |
| 17 | Fachinetti et al(57) | 2018 | 76 | F | Right Abdominal wall | Invasive lobular adenocarcinoma | ER+, PR+, HER2- | Y | N | Y | Y | N | N | F/up clinically 3mothly. Recurrence at 19mo. |
| 18 | Loh et al(58) | 2019 | 47 | F | Right inguinal | Carcinoma favouring breast differentiation | ER+, PR+ | - | N | Y | Y | Y | - | - |
| 19 | Rodriguez et al(59) | 2019 | 61 | F | Left chest wall | Invasive ductal adenocarcinoma | ER-, PR-, HER2+ | N | - | Y | - | - | - | - |
| 20 | Thasanabanchong et al(60) | 2020 | 51 | F | Left costal ridge | Invasive ductal adenocarcinoma | ER-, PR+, HER2- | N | N | Y | Y | Y | Y | F/up clinically + mammogram, no recurrence at 2yr. |
| 21 | Fernandez et al(61) | 2020 | 78 | F | Left chest wall | Invasive ductal adenocarcinoma | ER+, PR+ | Y | N | N | - | - | Y | Reduced tumour size after 8mo of systemic therapy, awaiting maximal response before surgery |
| 22 | Byon et al(62) | 2021 | 65 | M | Suprapubic | Invasive ductal carcinoma | ER+, PR+, HER2+ | Y | Y | Y | Y | - | Y | F/up with 3monthly CEA, CTTAP + bone scan every 3-5months. Recurrence at 3yrs. Ongoing f/u with CTTAP 3 monthly and bone scan 5monthly |
| 23 | Bansal et al(63) | 2022 | 69 | M | Left inguinal | Invasive ductal adenocarcinoma | ER+, PR+, HER2- | N | N | Y | - | Y | Y | F/up PET at 6mo, no recurrence |
| 24 | Cicek et al(64) | 2023 | 67 | F | Right inguinal | Invasive ductal adenocarcinoma | ER+, PR+, HER2- | N | Y | Y | Y | Y | Y | F/up at 6mo with DCMRI & PETCT |
| 25 | Nie et al(65) | 2024 | 63 | M | scrotum | Invasive adenocarcinoma of ectopic breast | ER-, PR-, HER2- (Triple negative) | Y | Y | Y | Y | - | N | Recurrence at 15mo, died at 17mo |
| 26 | Barrios et al(66) | 2024 | 60 | F | Left inguinal | Invasive ductal adenocarcinoma with mucinous differentiation | ER+, PR-, HER2- | N | N | Y | Y | Y | Y | No recurrence up to 3yr |

**Footnote and abbreviations: F**, female;**M,**male;**ER**, oestrogen receptor; **PR**, progesterone receptor; **HER2**, human epidermal growth factor receptor 2; **+/−**, positive/negative receptor status; **Y**, yes; **N**, no; **−**, not mentioned in article; **N/A**, not applicable; **Mo**, month; **Yr**, year; **F/up**, follow-up; **Lymph node and** **Organ metastasis**, includes metastases present at diagnosis and at recurrence (recurrence time frame is specified when applicable)

Supplementary Table S4: JBI critical appraisal scores for each included study across all checklist items.

| **Study Reference** | **Year** | **Were patient’s demographic characteristics clearly described?** | **Was the patient’s history clearly described and presented as a timeline?** | **Was the current clinical condition of the patient on presentation clearly described?** | **Were diagnostic tests or assessment methods and the results clearly described?** | **Was the intervention(s) or treatment procedure(s) clearly described?** | **Was the post-intervention clinical condition clearly described?** | **Were adverse events (harms) or unanticipated events identified and described?** | **Does the case report provide takeaway lessons?** | **Adjusted Score** | **Risk of bias** |
| --- | --- | --- | --- | --- | --- | --- | --- | --- | --- | --- | --- |
| Greene et al | 1935 | N | Y | Y | Y | Y | N | N | Y | 5.0 | Moderate |
| Simon et al | 1988 | N | Y | Y | Y | Y | Y | Y | Y | 7.0 | Low |
| Rose et al | 1990 | N | Y | Y | Y | Y | Y | Y | Y | 7.0 | Low |
| Livesey et al | 1990 | Y | N | Y | Y | Y | N | N | Y | 5.0 | Moderate |
| Bailey et al | 1993 | N | Y | Y | Y | Y | Y | U | Y | 6.5 | Moderate |
| Levin et al | 1995 | Y | Y | Y | Y | Y | Y | Y | Y | 8.0 | Low |
| Dyess et al | 1995 | Y | Y | Y | Y | Y | N | N | Y | 6.0 | Moderate |
| Kennedy et al | 1996 | Y | Y | Y | Y | Y | Y | Y | Y | 8.0 | Low |
| Irvin et al | 1999 | Y | Y | Y | Y | Y | N | N | Y | 6.0 | Moderate |
| Castro et al | 2001 | Y | Y | Y | Y | U | N | N | N | 4.5 | High |
| Rho et al | 2001 | N | U | U | Y | U | N | N | Y | 3.5 | High |
| Chung-Park et al | 2002 | Y | Y | Y | Y | Y | Y | Y | Y | 8.0 | Low |
| Roorda et al | 2002 | Y | N | Y | N | U | N | N | Y | 3.5 | High |
| Yin et al | 2003 | Y | Y | Y | Y | Y | N | N | U | 5.5 | Moderate |
| Tanaka et al | 2005 | U | U | Y | Y | Y | Y | Y | Y | 7.0 | Low |
| Lopes et al | 2006 | Y | Y | Y | Y | Y | N | U | Y | 6.5 | Moderate |
| Fracchioli et al | 2006 | U | Y | Y | Y | Y | Y | Y | Y | 7.5 | Low |
| Abbott et al | 2006 | Y | Y | Y | Y | Y | Y | Y | Y | 8.0 | Low |
| Kazakov et al | 2006 | U | U | Y | U | U | N | U | Y | 4.5 | High |
| North et al | 2007 | U | Y | Y | Y | Y | Y | N/A | Y | 6.5 | Low |
| Martinez-palones et al | 2007 | N | N | U | Y | Y | N | N | Y | 3.5 | High |
| van Herwaarden-Lindeboom et al | 2007 | Y | Y | Y | U | U | N | N | Y | 5.0 | Moderate |
| da Silva et al | 2008 | Y | Y | Y | Y | Y | N | Y | Y | 7.0 | Low |
| Kahraman-cetintas et al | 2008 | Y | Y | Y | U | Y | N | N | Y | 5.5 | Moderate |
| Ogino et al | 2010 | Y | N | Y | Y | Y | N | N | Y | 5.0 | Moderate |
| Naseer et al | 2011 | Y | Y | Y | Y | Y | Y | N | Y | 7.0 | Low |
| Rajan et al | 2011 | U | U | Y | Y | Y | Y | Y | U | 6.0 | Moderate |
| da Costa et al | 2012 | Y | Y | Y | Y | Y | Y | Y | Y | 8.0 | Low |
| Benito et al | 2013 | U | N | Y | Y | Y | Y | Y | Y | 6.5 | Moderate |
| Bogani et al | 2013 | Y | U | Y | Y | Y | Y | Y | Y | 7.0 | Low |
| McMaster et al | 2013 | Y | Y | Y | Y | Y | Y | Y | Y | 8.0 | low |
| Lamb et al | 2013 | Y | Y | Y | Y | Y | Y | Y | Y | 8.0 | Low |
| Francone et al | 2013 | U | U | Y | Y | Y | Y | N | Y | 6.0 | Moderate |
| Cripe et al | 2015 | Y | N | Y | Y | Y | Y | Y | Y | 7.0 | Low |
| Xu et al | 2015 | Y | Y | Y | Y | Y | Y | N | Y | 7.0 | Low |
| Samanta et al | 2015 | Y | Y | Y | U | U | Y | Y | Y | 7.0 | Low |
| Kopanakis et al | 2016 | Y | Y | N/A | Y | Y | Y | Y | Y | 7.0 | Low |
| Serre et al | 2016 | Y | Y | Y | Y | Y | Y | Y | Y | 8.0 | Low |
| Kredentser et al | 2016 | Y | N | Y | Y | Y | Y | Y | Y | 7.0 | Low |
| Kredentser et al | 2016 | Y | N | Y | Y | Y | Y | Y | Y | 7.0 | Low |
| Kredentser et al | 2016 | Y | N | Y | Y | Y | Y | N/A | Y | 6.0 | Low |
| Ishigaki et al | 2017 | Y | Y | U | Y | Y | Y | N | Y | 6.0 | Moderate |
| Eom et al | 2017 | Y | N | Y | Y | U | U | Y | Y | 6.0 | Moderate |
| Al-Mansouri et al | 2018 | Y | Y | Y | Y | Y | Y | Y | Y | 8.0 | Low |
| Lopes et al | 2018 | Y | Y | Y | Y | Y | Y | U | Y | 7.0 | Low |
| Zhong et al | 2018 | U | U | Y | Y | U | N | Y | Y | 5.5 | Moderate |
| Fachinetti et al | 2018 | Y | Y | Y | Y | Y | Y | Y | Y | 8.0 | Low |
| Loh et al | 2019 | U | U | Y | Y | Y | N | N | U | 4.5 | High |
| Rodriguez | 2019 | U | U | Y | Y | U | N | N | N | 3.5 | High |
| Matak et al | 2020 | U | Y | Y | Y | Y | Y | Y | Y | 7.5 | Low |
| Ananthula et al | 2020 | U | Y | Y | Y | Y | Y | Y | Y | 7.5 | Low |
| Thasanabanchong et al | 2020 | Y | Y | Y | Y | U | Y | Y | Y | 7.5 | Low |
| Fernandez et al | 2020 | U | Y | Y | Y | Y | Y | Y | Y | 7.5 | Low |
| Xie et al | 2021 | U | N | Y | Y | Y | Y | Y | N | 5.5 | Moderate |
| Byon et al | 2021 | U | U | Y | Y | Y | N | Y | Y | 6.0 | Moderate |
| Kovacs et al | 2022 | U | Y | Y | N | Y | Y | Y | Y | 6.5 | Moderate |
| Bansal et al | 2022 | U | U | Y | Y | Y | Y | Y | Y | 7.0 | Low |
| Sandak et al | 2023 | Y | Y | Y | Y | Y | N | N | N | 5.0 | Moderate |
| Cicek et al | 2023 | Y | Y | Y | Y | Y | Y | Y | Y | 8.0 | Low |
| Aksu et al | 2024 | Y | Y | Y | Y | Y | N/A | N/A | Y | 6.0 | Low |
| Tang et al | 2024 | U | N | U | N | Y | N | N | Y | 3.0 | High |
| Nie et al | 2024 | Y | Y | Y | Y | U | Y | Y | Y | 7.5 | Low |
| Barrios et al | 2024 | U | Y | Y | Y | Y | Y | Y | Y | 7.5 | Low |

Legend:
Y = Yes (1 mark), U = Unclear (0.5 mark), N = No (0 mark), N/A= Not applicable. Risk of bias was determined based on adjusted scores: Low risk = score ≥ 7, Moderate risk = score 5–6.5, High risk = score ≤ 4. Scores with 0.5 values were not rounded up for classification purposes.
